# Bacterial pore-forming toxins

**DOI:** 10.1099/mic.0.001154

**Published:** 2022-03-25

**Authors:** Fatima R. Ulhuq, Giuseppina Mariano

**Affiliations:** ^1^​ Microbes in Health and Disease Theme, Newcastle University Biosciences Institute, Newcastle University, Newcastle upon Tyne, UK

**Keywords:** pore-forming, bacterial toxin, virulence factor, bacterial antagonism

## Abstract

Pore-forming toxins (PFTs) are widely distributed in both Gram-negative and Gram-positive bacteria. PFTs can act as virulence factors that bacteria utilise in dissemination and host colonisation or, alternatively, they can be employed to compete with rival microbes in polymicrobial niches. PFTs transition from a soluble form to become membrane-embedded by undergoing large conformational changes. Once inserted, they perforate the membrane, causing uncontrolled efflux of ions and/or nutrients and dissipating the protonmotive force (PMF). In some instances, target cells intoxicated by PFTs display additional effects as part of the cellular response to pore formation. Significant progress has been made in the mechanistic description of pore formation for the different PFTs families, but in several cases a complete understanding of pore structure remains lacking. PFTs have evolved recognition mechanisms to bind specific receptors that define their host tropism, although this can be remarkably diverse even within the same family. Here we summarise the salient features of PFTs and highlight where additional research is necessary to fully understand the mechanism of pore formation by members of this diverse group of protein toxins.

## Introduction

In both eukaryotic and prokaryotic cells, the plasma membrane comprises two leaflets of amphipathic phospholipids, which form a physical barrier separating the cytoplasm from the extracellular milieu. The membrane is selectively permeable, through the action of transmembrane proteins that are involved in various processes that span from signal transduction to ion and nutrient transport [1]. Biological membranes often provide the first line of defence against external assaults, and are the targets of some of the most potent antibiotics and protein toxins [2]. Pore-forming toxins (PFTs) are key proteinaceous agents that target the plasma membrane, and they form the largest class of bacterial protein toxins [3–5] (Tables 1 and 2). In this review we summarise the variety of PFTs produced by bacteria, describing the mechanism of pore-formation, where known, as well as recent advances in the determination of their mode of action and the cellular responses they elicit in both their eukaryotic and prokaryotic targets. In particular, we review for the first time the diverse groups of anti-prokaryotic PFTs that have recently been identified.

**Table 1.** List of bacterial pore-forming toxins with anti-eukaryotic targets. For definition of class see text

Pore-forming toxin	Family	Class (Where known)	Producing organism	Receptor (Where known)
ClyA (HlyE)	ClyA	α	*E. coli, Salmonella* spp. * Shigella* spp.	Cholesterol [62]
Non-hemolytic tripartite enterotoxin (Nhe)	ClyA	α	* B. cereus *	Cholesterol [62]
Haemolysin BL (Hbl)	ClyA	α	* B. cereus *	Cholesterol, LITAF, CDIP1 [52]
YaxAB	ClyA	α	* Y *. *enterocolitica *	–
AhlABC	ClyA	α	* A. hydrophila *	–
SmhABC	ClyA	α	* S. marcescens *	–
MakABE	ClyA		* V. cholerae *	–
Aerolysin	Aerolysin	β	* A. hydrophila *	GPI-anchored proteins (CD52), Thy-1 [33, 45]
α-toxin	Aerolysin	β	* C. perfringens *	GPI-anchored proteins (CD52), folate receptor [33, 45]
ε-toxin (Etx)	Aerolysin	β	* C. perfringens *	HAVCR1, MAL [36, 85]
Enterotoxin (CPE)	Aerolysin	β	* C. perfringens *	Claudin [228]
Monalysin	Aerolysin	β	* P. entomophila *	–
α-haemolysin (Hla)	Haemolysin	β	* S. aureus *	Phosphatidylcholine/Sphingomyelin/ADAM10 [46, 229]
γ-haemolysin (Hlg)	Haemolysin	β	* S. aureus *	Phosphatidylcholine [230]
LukED	Haemolysin	β	* S. aureus *	CXCR1 and CXCR2 Duffy antigen receptor for chemokines (DARC) [231, 232]
LukAB	Haemolysin	β	* S. aureus *	CD11b/ HVCN1 [51]
HlgACB	Haemolysin	β	* S. aureus *	CXCR1, CXCR2, CCR2, C5aR and C5L2 [233]
Panton-Valentine Leukocidin (PVL)	Haemolysin	β	* S. aureus *	C5aR and C5L2 [48, 49]
β-toxin	Haemolysin	β	* C. perfringens *	PECAM-1 [47]
Necrotic enteritis toxin B (NetB)	Haemolysin	β	* C. perfringens *	Cholesterol [91]
δ toxin	Haemolysin	β	* C. perfringens *	Monosialic ganglioside 2 (GM2) [234]
* V. cholerae* cytolysin (VCC)	Haemolysin	β	* V. cholerae *	Glyco-conjugates [42, 43]
* V. vulnificus* cytolysin (VVH)	Haemolysin	β	* V. vulnificus *	Glycerol, N-acetyl-d-galactosamine [44]
Perfringolysin O (PFO)	CDCs	β	* C. perfringens *	Cholesterol, glycans [38, 59]
Suilysin (SLY)	CDCs	β	* S. suis *	Cholesterol, glycans [38, 59]
Intermedilysin (ILY)	CDCs	β	* S. intermedius *	Cholesterol, CD59, N-linked glycan [38–41]
Listeriolysin O (LLO)	CDCs	β	* L. monocytogenes *	Cholesterol, glycans [38, 59]
Lectinolysin (LLY)	CDCs	β	* S. mitis *	Cholesterol, CD59, glycans [38, 41]
Anthrolysin O (ALO)	CDCs	β	* B. anthracis *	Cholesterol, glycans [38, 59]
Streptolysin O (SLO)	CDCs	β	* S. pyogenes *	Cholesterol, glycans [38, 59]
Pneumolysin (PLY)	CDCs	β	* S. pneumoniae *	Cholesterol, glycans [38, 59]
Vaginolysin (VLY)	CDCs	β	* G. vaginalis *	Cholesterol, glycans [38, 59]
α-haemolysin (HlyA)	RTX	–	* E. coli* UPEC	CD11/CD18, glycophorin [53, 54]
EhxA	RTX	–	* E. coli* EAEC	–
Adenylate cyclase-haemolysin toxin (CyaA)	RTX	–	* B. pertussis *	CD11/CD18, CR3 [53, 55]
LktA	RTX	–	* M. haemolytica *	CD11/CD18 [53]
PlLktA	RTX	–	* M. varigena *	–
LtxA	RTX	–	* A. actinomycetemcomitans *	CD11/CD18 [53]
PaxA	RTX	–	*P. aerogenes*	CD11/CD18 [53]
PvxA	RTX	–	* P. vulgaris *	–
MmxA	RTX	–	*M. morganii*	–
ApxIA	RTX	–	*A. pleuropneumoniae*	–
ApxIIA	RTX	–	*A. pleuropneumoniae*	–
VcRtxA	RTX	–	* V. cholerae *	–
MARTX	RTX	–	* A. hydrophila *	–
MARTX	RTX	–	* V. cholerae *	–
VopQ	–	α	* Vibrio* spp.	c-ring subunit of V_o_ subcomplex of V-ATPase [147]
ShlA	T5SS-secreted haemolysin	–	* S. marcescens *	–
ExlA	T5SS-secreted haemolysin	–	* P. aeruginosa *	–
HpmA	T5SS-secreted haemolysin	–	*Proteus mirabilis,* * Proteus vulgaris *	–
EthA	T5SS-secreted haemolysin	–	* Edwardsiella tarda *	–
HecA	T5SS-secreted haemolysin	–	* Erwinia chrysanthemi *	–
HhdA	T5SS-secreted haemolysin	–	* Haemophilus ducreyi *	–

**Table 2.** List of bacterially-produced anti-bacterial pore-forming toxins

Toxin	Class (Where known)	Producing organism	Immunity (Where known)	Receptor (Where known)	Import
Colicin A	α	*Citrobacter freudii*	Cai	BtuB [235]	OmpF, TolABQR
Colicin B	α	* E. coli *	Cbi	FepA [236]	TonB-ExbBD
Colicin E1	α	* E. coli *	Cei	BtuB [237]	TolC, TolAR
Colicin Ia	α	* E. coli *	Iia	Cir [238]	TonB-ExbBD
Colicin Ib	α	* E. coli *	Imm	Cir [238]	TonB-ExbBD
Colicin K	α	* E. coli *	Cki	Tsx [239]	OmpF, TolABQR
Colicin N	α	* E. coli *	Cni	LPS/OmpF [168, 170]	OmpF,TolAQR
Colicin S4	α	* E. coli *	Csi	OmpW [240]	OmpF, TolABQR
Colicin U	α	* Shigella boydii *	Cui	OmpA [241]	OmpF,TolABQR
Colicin 5	α	* E. coli *	Cfi	Tsx [242]	TolC, TonB-ExbBD
Colicin 10	α	* E. coli *	Cti	Tsx [243]	TolC,TonB-ExbBD
Colicin 28b	α	* S. marcescens *	–	OmpA, OmpF, LPS [244]	OmpF, TolABQR
CdiA-CT^EC93^	–	* E. coli *	CdiI^EC83^	–	CdiB^EC93^
Pyocin S5	α	* P. aeruginosa *	ImS5	CPA [162]	FtpA/TonB
VasX	–	* V. cholerae *	TsiV2	–	T6SS
Tme1	-	* V. parahaemolyticus *	Tmi1	–	T6SS
Tme2	-	* V. parahaemolyticus *	Tmi2	–	T6SS
Tse4	–	* P. aeruginosa *	Tsi4	–	T6SS
Ssp6	–	* S. marcescens *	Sip6	–	T6SS
TspA	–	* S. aureus *	TsaI	–	T7SS

It should be noted that while PFTs are also produced by archaea and eukaryotes, this review will focus solely on bacterial toxins; for readers interested in PFTs produced by these other systems they are referred to the following articles [3–5].

## Mechanism of pore-formation

The first fundamental step of pore formation consists of binding of toxin protomers to a receptor on the surface of the target cell membrane. Receptors can be lipids, glycans or proteins. Receptor binding generally has two functions; it serves to increase the local concentration of the toxin, and also to promote oligomerisation [6–8].

Pore-forming toxins can be divided into two classes: α-PFTs and β-PFTs, according to whether the structure adopted by their membrane-spanning region is constituted of α-helices or amphipathic β-strands [3].

**Fig. 1.**
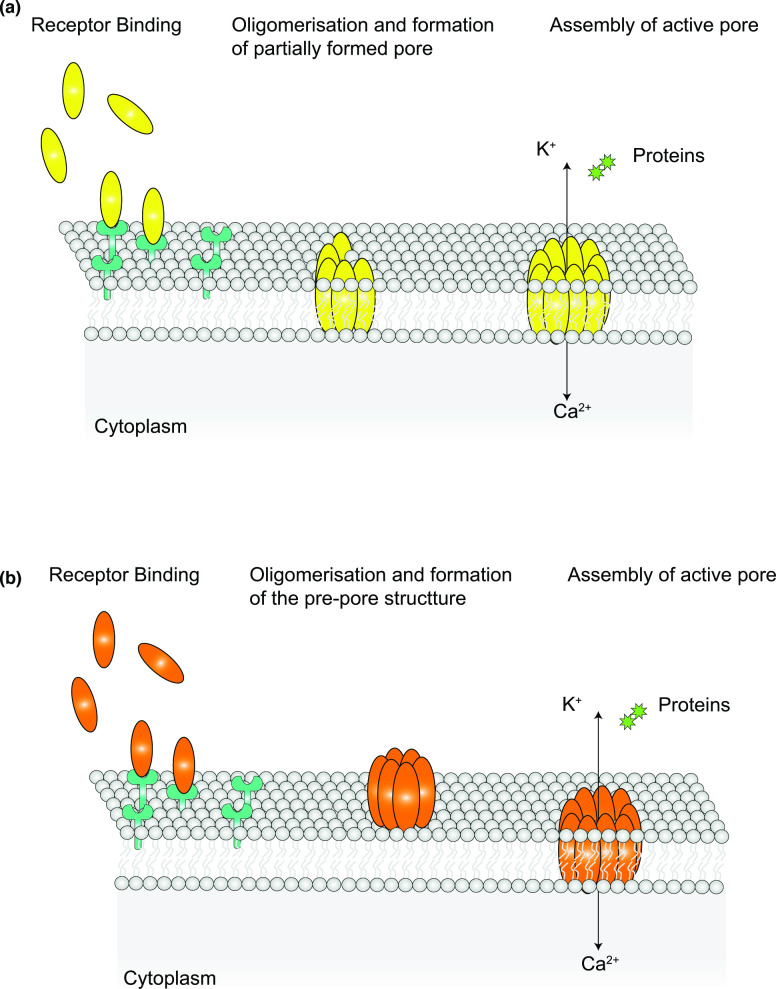
Schematic representation of the alternative pore forming mechanisms for α− and β−PFTs. (**a**) Most α−PFTs bind to specific receptors and once a critical concentration is reached PFT subunits insert concomitantly into the membrane and oligomerise to form the final pore. This mechanism of pore formation can sometimes result in formation of an incomplete pore that, none-the-less, retains function. (**b**) Protomers of most β−PFTs instead, following their concentration at the membrane interface, accumulate into a structure known as the pre-pore. Once oligomerisation is complete, the pre-pore subunits undergo massive conformational change to concertedly insert into the membrane.

Oligomerisation generally proceeds differently for α- and β-PFTs. For most α-PFTs, the monomers undergo a structural change that exposes their hydrophobic/amphipathic helices to a hydrophilic environment, promoting them to partition into the membrane (Fig. 1a). Consequently, oligomerisation and membrane insertion of α-PFTs is often concomitant. This results in a more flexible molecular organisation of α-PFTs, which renders this class of toxins quite heterogeneous in their structure. Usually, α-PFTs, such as cytolysin A (ClyA) and fragaceatoxin C (FraC) form pores whereby the protomers create a closed ring, which is then able to perforate the membrane [3, 8–11]. However, many α-PFTs are also reported to form partial pores, where the ring is incomplete, or toroidal pores, with a lumen consisting of both protein segments and lipids. Nevertheless, despite their ‘incomplete’ structure, these pores retain functionality [3, 12–14].

In the alternative pathway, commonly adopted by β-PFTs, the oligomerisation step occurs first, with sequential addition of monomers at the membrane interface to generate an intermediate structure, called the pre-pore (Fig. 1b). The pre-pore then undergoes conformational changes that favours its insertion into the membrane [15–17]. In the final, active pore, one or more β-strands from each monomer contribute to the formation of a β-barrel that spans the membrane. Within the β-barrel structure, a series of hydrogen bonds are established between amino acid sidechains of distinct monomers, ultimately conferring high structural rigidity and stability to the pore [15, 18, 19]. For some cholesterol-dependent cytolysins (CDCs) it was observed that the process of sequential addition of monomers to the pre-pore structure remains incomplete, leading to formation of arcs, as well as complete pores. These arcs display lower stoichiometry but remain active once inserted into the membrane [20].

PFTs can be further divided into different families (Tables 1 and 2), classified according to sequence and structural similarity of the pore and the presence of a conserved mechanism of membrane insertion [3, 4]. The stoichiometry and, thus, final lumen size of pores formed by members of different families can vary significantly. PFTs can range from monomeric pores that cause small membrane lesions to very large pores, such as those observed for CDC toxins, composed of 30–50 subunits (Tables 1 and 2).

In both cases, following insertion, hydrophilic residues are exposed in the lumen of the pore region, favouring the influx/efflux of ions, proteins and nutrients, whereas the hydrophobic regions are exposed towards the fatty acid tails in the bilayer [19]. In some cases, PFTs exhibit a preferred permeability towards specific ions. Typically, the ability of pores to discriminate between different ions is determined by selectivity filters. Selectivity is dictated by the nature of the amino acids that line the narrowest part of the pore lumen and that specifically interact with the transported ions [21].

X-ray crystallography and cryo-electron microscopy (cryo-EM) have been extensively used to determine the structural changes that PFTs undergo when transitioning from monomers to fully assembled pores. Where the generation of high-resolution structural information has not been possible, alternative approaches have been used to characterise the nature of the PFT fold, conformational changes and membrane insertion. These include atomic force microscopy (AFM), small-angle X-ray scattering (SAXS), *in silico* structural prediction, nuclear magnetic resonance (NMR) and electron paramagnetic resonance (EPR) spectroscopy. For many of these approaches, proteoliposomes and artificial lipid bilayers have been used to facilitate investigation of PFTs in the context of a lipid-rich environment, by mimicking the target membrane. Finally, studies with lipid bilayers in patch-clamping conditions and permeabilisation of lipid vesicles to fluorescent compounds of different sizes, have allowed PFT selectivity and pore diameter to be defined. In the following sections, we summarise how these approaches have advanced our understanding of the mechanism of PFT assembly for each toxin family.

## Pore forming toxins as virulence factors

Many PFTs represent virulence factors and play multifaceted roles in pathogen infection, by directly or indirectly contributing to pathogen invasion and dissemination [22, 23]. Pore insertion into the eukaryotic plasma membrane causes uncontrolled efflux of nutrients and ions, especially K^+^, and can also perturb Ca^2+^ signalling. Various bacterial secretion systems have been linked with the secretion of anti-eukaryotic PFTs including the type I (T1SS), type II (T2SS), type III (T3SS) and type V secretion systems (T5SS) and the general secretory (Sec) pathway [24–29]. Outer membrane vesicle release has also been implicated in toxin delivery [30].

A significant number of anti-eukaryotic PFTs have been identified and characterised to date, and have been classified into different families based on structural features and mode of action: these are summarised in Table 1. While the vast majority of PFTs that target eukaryotes are produced by bacterial species, some members of the actinoporin family have only been found in sea-anemone species, and certain PFTs in the aerolysin family are also found in Cnidaria, fungi and earthworms [3]. It is worth noting that most of the PFTs identified to date are produced by Firmicutes and Proteobacteria (Table 1). It remains to be seen whether future studies will identify PFTs in other, less studied phyla.

### Receptors for anti-eukaryotic PFTs

PFTs interact with target membranes with very high specificity. PFTs belonging to the same family can bind to different membrane receptors. In other cases, one PFT exhibits the ability to target several cell types through binding to different receptors [3, 4, 31, 32]. It is therefore not surprising that many studies have found that PFTs belonging to the same class differ in the receptors they bind to because they interact with distinct structural motifs. This influences the specific types of cells that they target, thus expanding the cellular tropism of a single class [3, 4]. In several cases, the attachment of PFT monomers to the membrane can involve binding to more than one type of receptor (Table 1) [33–37].

#### PFT specificity for glycan receptors

For many PFTs, binding to the target membrane is via surface glycans. These are typically glycans that are conjugated to membrane proteins although, in some instances, PFTs bind to protein glycosyl phosphatidyl inositol (GPI) membrane anchors [3, 4].

All members of the CDC family have been shown to display lectin activity, and to bind glycans that are conjugated to cellular receptors for the initiation of pore formation (Table 1). In some instances, for example perfringolysin O (PFO) and streptolysin O (SLO), the CDCs do not show high selectivity and can interact with multiple classes of glycans, whereas pneumolysin (PLY), lectinolysin (LLY), intermedilysin (ILY) and listeriolysin O (LLO) only bind to a single distinct class of glycans [38]. ILY from *Streptococcus intermedius* was shown to interact with the GPI-anchored erythrocyte receptor CD59 through its sialyl-TF O-glycan [38–41].

The haemolysin *Vibrio cholerae* cytolysin (VCC) was shown to bind glycosylated protein receptors at the membrane interface through its β-trefoil and β-prism domains (Table 1) [42, 43]. *Vibrio vulnificus* haemolysin (VVH), from the same class, also employs the β-trefoil domain to recognise and bind a broader spectrum of galactosyl groups, including N-acetyl-d-galactosamine (GalNAc) and N-acetyl-d-lactosamine (LacNAc), with micromolar affinity (Table 1) [44].

*Aeromonas hydrophila* aerolysin employs an elongated N-terminal domain to interact both with N-linked glycans and the GPI anchor of its different receptors (Table 1) [33, 34]. Aerolysin binds the second mannose on GPI anchors and mannose modification or removal results in resistance to the toxin [35]. Similarly, another aerolysin homologue, *Clostridium septicum* α-toxin was also found to interact with its receptors via their GPI anchors [36]. Interestingly, Gordon *et al*. found that despite binding to GPI anchors, aerolysin and α-toxin display different affinities for different receptors, with aerolysin binding more strongly to the Thy-1 receptor, whereas α-toxin showed higher affinity for the folate receptor [45]. Despite the existing evidence that aerolysin-like toxins bind to receptors via their glycan modifications, many gaps in knowledge still exist. For example, while *Clostridium perfringens* ε-toxin was found to bind hepatitis A virus cellular receptor 1 (HAVCR1), an O-linked glycoprotein, the role of the sugar moieties of HAVCR1 in this interaction is yet to be established (Table 1) [36].

#### PFT specificity for protein receptors

PFTs can also recognise protein receptors for membrane attachment. Furthermore, the ability of certain toxins to recognise specific protein motifs, in addition to sugar moieties, allows many PFTs to display a diversified cellular tropism, as shown for aerolysin and α-toxin [45].

*Staphylococcus aureus* α-haemolysin (Hla) can target epithelial cells through binding to disintegrin and metalloproteinase domain-containing protein 10 (ADAM10) (Table 1) [46], whereas the β-toxin from *C. perfringens* specifically interacts with CD31 and platelet endothelial cell adhesion molecule-1 (PECAM-1) receptors (Table 1), allowing targeted infection of endothelial cells [47]. Additionally, the *S. aureus* Panton-Valentine leukocidin (PVL) specifically targets neutrophils by interacting with C5a receptor 1 (C5aR1) and C5a receptor-like 2 (C5L2) (Table 1) [48, 49]. Interactions with these receptors allow PVL to also target, albeit with lower efficiency, monocytes and macrophages [48].

Leukocidin AB (LukAB) shows tropism for human phagocytes through binding with the integrin component CD11b (Table 1) [37, 50]. In a recent study, it was found that polymorphisms exist in *lukAB* genes and that these seem to be highly dependent on *S. aureus* clonal complex (CC). Intriguingly, it was demonstrated that toxicity of two LukAB variants, produced by CC30 and CC45, did not depend on CD11b and that instead the receptor for these variants is the hydrogen voltage-gated channel 1 (HVCN1) [51]. Whilst the CC30 and CC45 variants still primarily targeted phagocytes, they also showed toxicity towards monocytes and neutrophils [51]. These findings highlight how genetic variability within the same toxin type could potentially further amplify the host tropism of toxins and increase its pathogenic effectiveness.

Haemolysin BL (Hbl), a tripartite toxin of the ClyA family (Table 1), binds to LPS-induced TNF-α factor (LITAF) and cell death involved p53 target 1 (CDIP1) [52]. LITAF is the primary receptor for Hbl, and its depletion greatly increases resistance to the toxin. CDIP1 and LITAF are homologues with highly-conserved C-terminal regions, however, CDIP1 can only act as an alternative receptor for Hbl at high toxin concentrations [52]. In support of its role as an alternative receptor, it was shown that a CDIP1 knock-out has no phenotype; however, a knock-out of both receptors confers complete resistance to Hbl [52].

HlyA and CyaA toxins, belonging to the Repeat-in-toxin (RTX) toxins, specifically bind to integrin heterodimer CD11/CD18, expressed on the surface of B and T cells, monocytes and neutrophils [53–55]. Interestingly, the same receptor has also been reported for other members of the same family, LtxA and LktA [54]. However, CyaA and HlyA can also bind to erythrocytes, causing haemolysis. Erythrocyte binding occurs in a receptor-independent manner for CyaA and through glycophorin for HlyA [31, 32]. Additionally, it was also observed that HlyA can bind to Nectin-2 in order to promote its interaction with kidney epithelial cells [56]. The diverse array of receptors that can be bound by distinct RTX toxins indicates that although these toxins may have developed specificity for binding to CD11/CD18 in order to effectively target and disrupt immune system cells, they still retain the ability to intoxicate other targets through electrostatic interaction with lipids or recognition of different receptors. This promiscuity provides a wider range of targets during the intoxication process.

#### PFT specificity for lipid receptors

Lipids can also act as specific receptors for PFTs, in particular when they are associated with lipid rafts or microdomains [57]. The best examples of PFTs recognising a lipid receptor are provided by CDCs, which bind to cholesterol to accumulate at the target membrane interface [3, 58]. PFO and SLO toxins both bind to cholesterol, and their oligomerisation can be modulated by cholesterol concentration in the target membrane [59]. Additionally, it was shown that cholesterol binding is mediated by a highly conserved peptide sequence within the CDC family, ECTGLAWEWWR [59]. Another study has demonstrated that PFO binding to cholesterol is further promoted by a threonine-leucine pairing at residues 490–491. The threonine-leucine pair is mostly conserved in the CDC family and could potentially play the same role for other CDCs [60]. However, NMR spectroscopy did not reveal any direct contact between cholesterol and this conserved pair for LLO [61]. Instead, Kozorog *et al.* demonstrated that Trp489 residue within the conserved sequence plays a role in pore formation whereas Trp512 is involved in membrane binding [61]. These observations highlight the need for further experimental work to define the mechanism of recognition and binding of CDCs to cholesterol, and to determine whether members of the same family employ different strategies for receptor recognition. This is particularly pertinent because all members of the CDC family also possess lectin activity and thus are able to bind glycan receptors (glyco-conjugated proteins or lipids) [38]. These findings further suggest that binding of CDCs to glycan receptors, ahead of their interaction with cholesterol, might facilitate a wide cellular and tissue tropism for these toxins and, in addition, could also provide a means to localise and concentrate CDCs to adjacent lipid rafts that contain cholesterol [38].

Binding to cholesterol is also a feature of the ClyA family. In particular, the N-terminal helix of ClyA binds to cholesterol, resulting in enhanced stability of the toxin intermediate states [62]. Molecular simulations showed that cholesterol binding could promote interaction between ClyA protomers, leading to pore formation [62]. Thus in this instance cholesterol does not represent a receptor for ClyA, but rather a mechanism to promote pore formation.

### Eukaryotic-targeting toxin families

As outlined in section 1, eukaryotic-targeting PFTs are grouped into different families, according to their mechanism of pore formation and structural similarity. In the following sections, the different families of eukaryotic-targeting PFTs are reviewed. For each PFT family, the complete diversity of identified members is listed in Table 1. Where information is available, the most recent structural and biophysical characteristics for members of each PFT family are discussed, including recent advances in the determination of their assembly, stoichiometry, and insertion mechanism into host cell membranes. Finally, for each PFT family, the impact of PFT-mediated intoxication on the physiology of the affected host cells is also reviewed.

#### The ClyA family

**Fig. 2.**
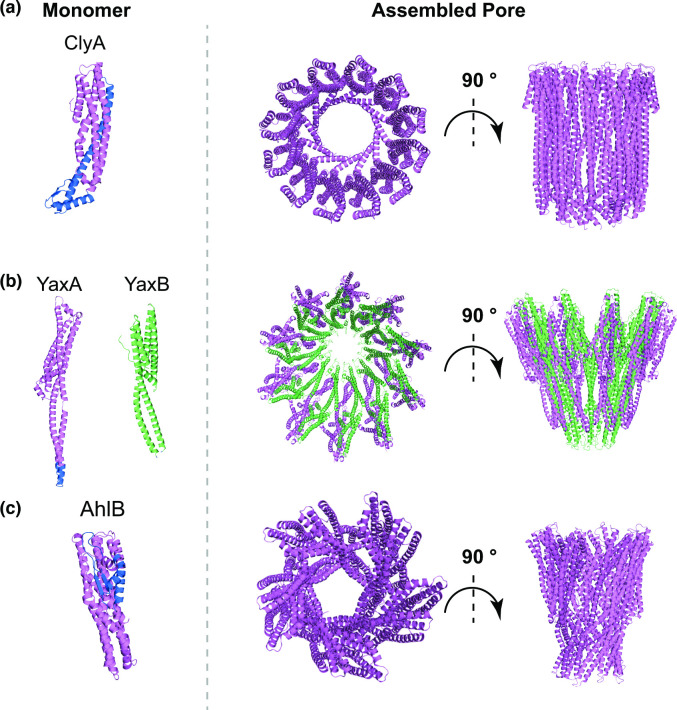
Cartoon representation of the molecular structure of the monomer (left), top and side view of the complete pore (right) for monopartite (**a**), bipartite (**b**) and tripartite (**c**) members of the ClyA family. In (**a**), PDB entry 1QOY was used to obtain the cartoon representation of ClyA monomeric structure and PDB entry 2WCD for its final pore structure. (**b**) The monomeric and oligomeric structure of bipartite toxin YaxAB are shown as representatives of a ClyA-family bipartite toxin. YaxA (PDBID: 6EK7) is shown in violet and YaxB (PDBID: 6EK8) in green. The YaxAB pore (PDBID: 6EL1) shows a distinct overall fold from monopartite ClyA. (**c**) Cartoon representation of the AhlB component of the tripartite toxin AhlABC is shown in its soluble monomeric form (PDB entry 6GRK) and as an assembled pore (PDB entry 6GRJ). While the core fold of AhlB pore remains closely related to ClyA and YaxAB, there are some differences in its overall architecture. For each panel, the membrane-spanning region in the monomer, where resolved, is shown in blue.

The ClyA family comprises α-PFTs. While many members of this family are made of a single type of protomer, some are bipartite (two different protomers) and even tripartite, comprising three different subunits (Table 1) [3]. The best characterised member of this family is ClyA, a single-component toxin found in some strains of *Escherichia coli *, *Salmonella enterica* and *Shigella flexneri* [8] (Table 1). The assembly of ClyA pores follows a sequential mechanism where single monomers are added consecutively to the assembling structure, leading to formation of the final pore [63]. ClyA monomers consist of mostly α-helices, except for a short region, defined as a β-tongue, which comprises a β-hairpin (Fig. 2a) [64]. Upon binding to the receptor, the β-tongue associates with the membrane, a process that is favoured by the hydrophobic nature of this region [64]. This results in major conformational changes of ClyA that ultimately allow the amphipathic α-helix at its N-terminus to contact the membrane surface. This α-helical region from each protomer subsequently forms a tightly packed helical barrel that is inserted into the membrane, with a final 12-mer stoichiometry (Fig. 2a) [64]. More recent work demonstrated that ClyA pores can also adopt tri-decamer and tetra-decamer stoichiometries, and confirmed that binding to cholesterol facilitates ClyA pore formation [65].

YaxAB from *Yersinia enterocolitica* is a bipartite member of the ClyA family (Table 1). The structures of monomeric YaxA and YaxB have been solved (Fig. 2b), revealing that they each possess an α-helical-rich domain with high structural similarity to ClyA, despite the low sequence conservation (Fig. 2b) [66]. Furthermore, YaxAB possesses two additional unique domains, a coiled-coil and a foot domain. In contrast to ClyA, the main stoichiometry found for YaxAB pores is a decamer of dimers (20-mer), and the assembled pore also shows a rather different architecture (Fig. 2b) [66]. The proposed model for YaxAB assembly suggests that YaxA initiates pore formation by binding to the membrane via its hydrophobic domain, thereby recruiting YaxB. Complex formation with YaxA induces major structural rearrangements in the YaxB foot domain, which transitions to a membrane-inserted state. Further oligomerisation proceeds through interactions between YaxAB heterodimers [66].

A tripartite member of the ClyA family, AhlABC, is found in *Aeromonas hydrophila* (Table 1). A model for AhlABC assembly has been proposed, whereby AhlC is initially found as a homotetramer, packing its hydrophobic residues inside a pore-like structure [9]. Upon disassembly of AhlC tetramers, AhlC monomers associate with one leaflet of the membrane, subsequently recruiting AhlB (Fig. 2b). The bound AhlB undergoes reorganisation involving refolding of a β-tongue region, as in ClyA, ultimately leading to formation of an elongated helical structure [9]. AhlA is also recruited during this process and the putative role for this protein is to provide a hydrophilic pore lining. Intermediate AhlB or AhlBC pores can also form and these display some activity, but the maximum lytic activity is found with fully formed AhlABC pores. In the assembled complex, AhlB forms the membrane-spanning region and the core AhlB pore exhibits a distinct fold compared to other members of the family (Fig. 2c) [9]. The crystal structures of SmhA and SmhB, components of the tripartite SmhABC toxin from *Serratia marcescens* (Table 1), have also been solved. This toxin is a homologue of AhlABC and accordingly, the structure of the SmhB pore displays close similarity to AhlB [10].

ClyA toxins cause lysis of red blood cells and, additionally, impair the Ca^2+^-based signalling pathways of intestinal epithelial cells [67]. It has also been shown that ClyA promotes apoptosis of macrophages/monocytes of both human and murine origin [68]. These observations highlight how ClyA may promote virulence of enteric pathogens, such as *E. coli* and *Salmonella.* Indeed *clyA* genes have been found in several strains of *E. coli *, including enteroinvasive strains and those producing shiga-toxin [69]. In *S. enterica* serovar Typhi, it was shown that *clyA* expression is enhanced after engulfment by macrophages and that the encoded toxin can lyse epithelial cells [70]. In *Salmonella* strains, ClyA appears to co-operate with the invasin TaiA in order to promote macrophage hijacking during chronic infection [70]. Conversely, *clyA* genes are not found in certain uropathogenic or enteropathogenic *E. coli* strains [69]. Furthermore, inactive ClyA variants are present in other enteric pathogens, such as *Shigella* strains [71], suggesting that ClyA’s role in pathogenesis might be limited to a subset of *Enterobacteriaceae* species. Moreover, even for those *E. coli* strains producing a functional ClyA homologue, the real impact and significance of this toxin in the pathogenicity process is unknown, as these strains produce numerous other toxins and virulence factors [69, 71, 72].

The tripartite toxins Nhe and Hbl (Table 1), both produced by *Bacillus cereus,* form pores in macrophages that lead to potassium efflux, thereby leading to cell death via the activation of the NOD-, LRR- and pyrin domain-containing 3 (NLRP3) inflammasome [73, 74]. Both toxins were shown to cause cell death in various human cell lines, in addition to macrophages and red blood cells. Similarly, YaxAB can cause both haemolysis and death of macrophages, however its specific significance in the pathogenesis of *Y. enterocolitica* has yet to be determined [66, 75]. Finally, the recently identified tripartite toxin MakABE, secreted by *V. cholerae *, was shown to cause Golgi fragmentation, depolarisation and rounding of mitochondria, disruption of actin filaments and depletion of cellular ATP [28].

#### The aerolysin family

The aerolysin family comprises a number of bacterial β-PFTs, including aerolysin from *Aeromonas* spp., α-toxin and ε-toxin from *C. perfringens* and monalysin from *Pseudomonas entomophila* [3] (Table 1).

**Fig. 3.**
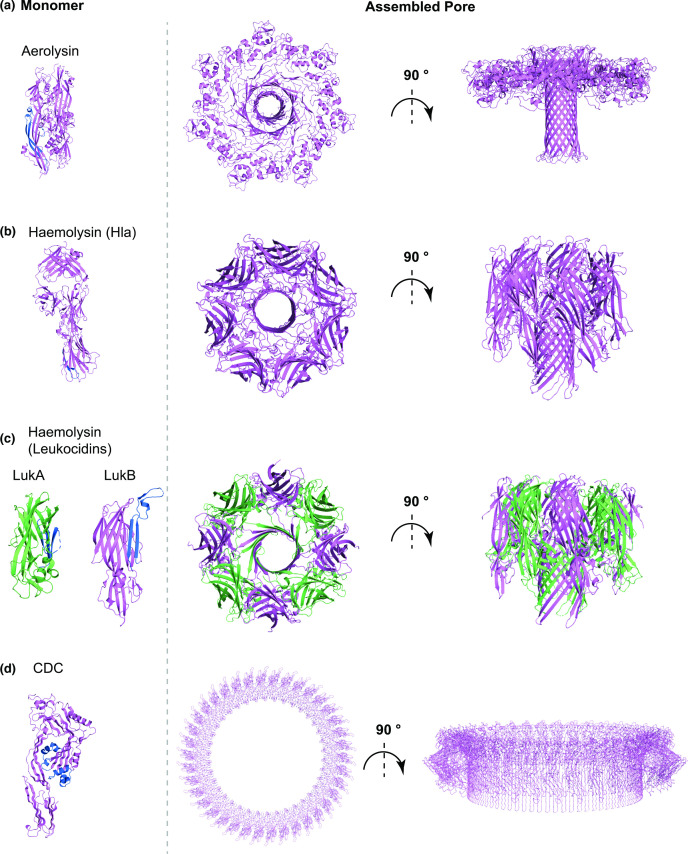
Cartoon representation of representative structures of the soluble monomer (left) and assembled pore (right) for (**a**) the aerolysin family, (**b**) the monopartite haemolysin family, (**c**) the bipartite haemolysin toxin family, and (**d**) the cholesterol-dependent cytolysin family (CDCs). (**a**) The aerolysin monomer (PBDID: 1PRE), shown on the left, undergoes massive rearrangements upon oligomerisation that lead to its extension and exposure of its β-barrel to the membrane. The β-barrel is then pushed into the membrane and its neighbouring region is rearranged in a rivet-like configuration for increased stability of the final pore (right, PDBID: 5JZT). (**b**) The Hla monomer (left, PDBID: 4IDJ) also undergoes conformational changes upon oligomerisation, similarly to aerolysin, that lead to extension of the β-barrel, which is then inserted into the membrane in the mature pore (PDBID: 7AHL). Whilst monomer extension is similar between aerolysin and Hla, the final pore structure as well as the lumen is quite different. In (**c**), the monomeric components LukA (PDBID: 5K59, green) and LukB (PDBID: 5K59, violet) of a bipartite leukocidin (haemolysin family) are shown on the left. Leukocidins conformation transition from soluble monomer to membrane-inserted form is similar to other haemolysins. The final assembled pore top and side views are shown on the right (PDBID: 4TW1) and it differs from Hla in terms of stoichiometry, whereas the overall 3D fold remains similar. (**d**) In the soluble monomer structure of perfringolysin O (left, PDBID: 1PFO) and other CDCs, the pore forming domain is organised as a β-sandwich between two a-helices. Electron microscopy and atomic force microscopy models show that in the membrane-inserted form, for each monomer, this region transitions to two amphipathic β-hairpins, which are inserted into the membrane, resulting in the final pore (biological assembly depiction from PDB entry 1PFO). For each panel, the membrane-spanning region in the monomer, where resolved, is show in blue.

Aerolysin is secreted in a soluble, monomeric form. The protein comprises several domains, including a receptor binding domain and a cleavable C-terminal region [76, 77]. Monomers are secreted in a pro-form, with the C-terminal region preventing premature oligomerisation [77]. Following C-terminal cleavage, aerolysin monomers associate into a heptameric pre-pore [34]. The core domain of aerolysin consists of a five stranded β-sheet and a pre-stem loop region (Fig. 3a), with the latter playing a crucial role in driving the conformational rearrangements that allow formation of the aerolysin pre-pore [34, 76].

Near-atomic structures of the different stages of aerolysin assembly have been solved, demonstrating that the pre-pore consists of two concentric β-barrel rings that sit at the membrane interface [34]. The pre-stem loop then folds within the pre-pore, triggering the conformational changes that allow transition to the mature pore (Fig. 3a) [34]. In the first step of aerolysin rearrangement, the inner β-barrel ring is extended towards the membrane, including the pre-stem loop. Subsequently, the extracellular portion of the protein is flattened towards the membrane, facilitating injection of the inner β-barrel into the membrane, in a piston-like mechanism [34]. Upon insertion into the membrane, hydrophobic regions found at the top of the inner β-barrel are repositioned in a rivet-like configuration that stabilises the pore within the membrane (Fig. 3a) [34].

The core structural feature that is typical of members of the aerolysin family is represented by the pre-stem loop and the two adjacent β-sheets (the DBB region) [3]. Sequence analysis and modelling has shown that this motif is conserved in all aerolysin members and that it is important to drive pore formation [78]. Both structural and bioinformatic studies also highlighted that although conservation of the primary sequence within aerolysin-like toxins might not always occur, other members of this family still retain the same structural features [78–80]. The importance of this region was further confirmed in a more recent study where the structure of the ε-toxin pore was determined [80]. Savva *et al.* showed that the ε-toxin structure essentially retains the same conformational features and fold of aerolysin and that the DBB region is pivotal to drive the pore conformational rearrangements that occur at the outer β-barrel and the membrane insertion process [80].

Aerolysin secretion contributes to *A. hydrophila *-induced sepsis and gastroenteritis [72]. Aerolysin triggers the NACHT- LRR- and PYD domain-containing protein (NALP3) inflammasome [81]. Furthermore, it was demonstrated that upon aerolysin pore formation, K^+^ efflux occurs, leading to a series of cellular responses that cause the reorganisation of tight junctions [82]. Additionally, pore formation results in accumulation of intracellular Ca^2+^, initiating a signalling pathway that culminates in myosin phosphorylation and actomyosin constriction [82]. Consequently, the redistribution of tight junctions further impairs the integrity of the intestinal epithelial barrier, favouring bacterial dissemination [82]. Perturbation of tight junction distribution and epithelial barrier integrity was also observed for *C. perfringens* ε-toxin and enterotoxin (CPE) [83, 84]. More recently, ε-toxin was also shown to selectively target and intoxicate specific subpopulations of lymphocytes that express myelin and lymphocyte (MAL) receptor (Table 1) [85]. Additionally, α-toxin from *C. perfringens* contributes to sepsis by promoting the host inflammatory response, through stimulation of cytokine production. Furthermore, α-toxin is able to perturb the host immune response by inactivating Granulocyte colony-stimulating factor (G-CSF) receptor, thereby preventing granulopoiesis and monocyte production [86].

#### The haemolysin family

Toxins in the haemolysin family are β-PFTs that are largely produced by *S. aureus* strains. Specifically, haemolysin members found in *S. aureus* can include both single component toxins, like α-haemolysin (Hla), and bipartite toxins, such as γ-haemolysin AB (HlgAB), HlgCB, leukocidin ED (LukED), Panton–Valentine leukocidin (PVL or LukSF) and leukocidin AB (LukAB; previously known as LukGH) (Table 1) [3, 87]. Other haemolysin toxins are also produced by other pathogens, such as *V. cholerae *, *V. vulnificus* and *C. perfringens* (Table 1) [3, 87].

The haemolysin family is one of the best characterised PFTs, and numerous near-atomic structures of soluble monomers and assembled pores have been determined (Fig. 3b) [88–94]. The soluble monomer of the single component toxin α-Hla is rich in β-strands and is formed from three domains; the stem, rim and β-sandwich domains [88]. In the monomeric structure, the stem domain is tightly packed against the rest of the protein (Fig. 3b). Following interaction with the membrane and heptamerisation, the stem domain of each protomer is rearranged away from the core of the protein, forming a partial β-barrel pre-pore. Finally insertion of the β-barrel into the membrane results in transition to the final active pore (Fig. 3b) [88]. The structural features and general fold of the α-Hla pore are highly conserved in both single component haemolysin toxins VCC and NetB [89, 91], and in bipartite toxins, such as LukAB, LukED and γ-haemolysin, although bipartite toxins form octameric rather than heptameric pores [88–93].

For bipartite haemolysins, the two toxin components were initially classified as S (slow) (LukS, LukE, HlgA and HlgC) and F (fast) (LukF, LukD, and HlgC), according to their elution on a chromatographic column [87]. S and F monomers display high structural similarity (Fig. 3c). Generally, the process of pore formation is initiated by binding of the S component to a cellular receptor. Subsequently the F component is recruited to the membrane, and oligomerisation is induced, thereby promoting the structural rearrangements that push the stem domain outwards to form the pre-pore [87]. The final octameric pore is constituted of four alternating subunits of each component, and the final fold is highly similar to that observed for Hla (Fig. 3c) [90, 92–94]. Intriguingly, the LukAB F component LukB shows the ability, albeit weak, to bind to receptor CD11b [92], and LukAB follows a slightly altered pathway to pore formation whereby the interaction between the two components LukAB takes places in their soluble monomeric state and heterodimer formation is necessary for efficient binding to CD11b and oligomerisation [92]. Furthermore, although the general fold of LukAB pore is similar to that of other bipartite haemolysins, several differences are found in both domain orientation and strength of intermolecular interactions compared to other leukocidins [92].

Despite the highly conserved structural organisation of members of the haemolysin family, these toxins can target different subtypes of immune cells through highly specific recognition of surface receptors [95, 96]. In addition to immune cells, Hla can also target and cause lysis of epithelial cells, thereby aiding bacterial dissemination during *S. aureus* infection [97]. LukAB and γ-haemolysin were both reported to target the NLRP3 inflammasome in monocytes and macrophages, by a mechanism that has yet to be fully elucidated. The current model suggests that K^+^ efflux causes NLRP3 inflammasome activation and a pro-inflammatory response, which leads to pyroptosis in a caspase-dependent manner [23, 96, 98]. Similarly, VCC was found to promote apoptosis in a caspase-dependent manner and vacuole formation in different human cell lines [99]. Finally, Hla was shown to induce filament-like structures in *S. aureus-*containing phagosomes, a process that was dependent on the presence of GTPases Rab1b, Rab7 and by the autophagic protein LC3 (100). Importantly, these filament-like structures were shown to be necessary for *S. aureus* replication in the phagosome, aiding its evasion of the innate immune system [100].

#### The cholesterol dependent cytolysin family

Members of the cholesterol dependent cytolysin (CDC) family are β-PFTs mainly produced by Gram-positive firmicute bacteria, including *Listeria *, *Clostridium *, *Bacillus* and *Streptococcus* spp [3]. (Table 1). To date, many studies have reported the structure of the soluble monomers of several members of the CDC family. The high-resolution structure of perfringolysin O (PFO) from *C. perfringens* was one of the first CDC structures reported, showing that the soluble monomer consists of an elongated structure rich in β-sheet (Fig. 3d) [101]. Subsequent studies have reported the structure of the soluble monomeric form of other members of the CDC family, including suilysin (SLY) from *Streptococcus suis* [102], SLO from *Streptococcus pyogenes* [103], ILY from *Streptococcus intermedius* [40], PLY from *Streptococcus pneumoniae* [104]*,* LLO from *Listeria monocytogenes* [105]*,* and vaginolysin (VLY) from *Gardnerella vaginalis* [41]. The structures reveal that all of these CDC monomers share a conserved fold, although they differ in the orientation of their C-terminal domains.

Despite an abundance of CDC monomer structures, atomic resolution detail of any pre-pore or pore structure is currently not available. Nevertheless, a combination of cryo-EM, AFM and molecular dynamics simulations have determined the fundamental steps that lead to the assembly of CDC pores [104, 106–108]. Interestingly, unlike aerolysins and haemolysins, where one β-sheet from each monomer constitutes the final pore structure, the final assembled pore of CDC toxins comprises two amphipathic β-hairpins from each protomer [104, 106–108]. In the soluble monomer, the region encompassing the amphipathic β-hairpins displays a different organisation, forming a β-sandwich flanked by two α-helices, an arrangement which prevents premature oligomerisation (Fig. 3d). During assembly, monomers are added sequentially to the assembling pre-pore, a process that has now been confirmed for several CDC toxins [107, 109, 110]. The use of a disulphide-locked SLY pre-pore coupled with real-time AFM demonstrated that normally pre-pore oligomerisation and assembly is completed before insertion into the membrane, a process also observed for PFO [107, 111]. Pre-pore and membrane insertion are then thought to trigger major conformational changes that result in α-helix-to-β-strand transition, forming the β-hairpin [104, 106–108]. During this process, the β-hairpins from each monomer expand and insert into the membrane [107]. These structural rearrangements have to take place in a concomitant manner in all subunits for pore assembly to be successfully completed [107].

Interestingly, it has been observed that in many cases the oligomerisation process is not completed and incomplete arcs with lower stoichiometry are also inserted into the membrane, which still retain their ability to form membrane lesions and active pores [20, 107]. For both fully formed pores or arcs, membrane insertion causes extrusion of lipids from the membrane, leading to formation of the final pore [107]. Additionally, AFM coupled with fluorescence microscopy demonstrated that transition from pre-pore to pore is characterised by a 40 Å collapse of the structure into the membrane [106]. Further modelling data have suggested that the collapse into the membrane could be generated by rotation of the central domain of the assembled pore, in a mechanism similar to that observed for aerolysin [34, 108]. Studies based on EM analysis and fitting of monomeric structures suggested that the β-hairpins that constitute the PLY β-barrel lie perpendicular to the membrane surface [104, 112]. PFO pores instead adopt a slightly modified arrangement where the β-hairpins display a 20° tilt with respect to the membrane, and this orientation is crucial for correct pore assembly and functionality [110]. CDC toxins form the largest known pores, with a final stoichiometry that varies, depending on each toxin, from 30 to 50 subunits (Fig. 3d) [3].

PFTs belonging to the CDC family share approximately 40% sequence similarity [113]. As previously discussed, the ECTGLAWEWWR sequence, which mediates binding to cholesterol, is highly conserved within this family [59]. Furthermore, recent work has highlighted that the motif F/Y-F/Y-Xn-YGR also displays a high degree of conservation within CDCs [114]. In particular, this motif is highly conserved in several proteins that share almost no other sequence similarity with known CDCs and are found in different bacterial species [114]. The X-ray structure of one of the newly identified proteins has been solved, highlighting that their 3D structure is nearly identical to monomers of different CDCs, defining these proteins as a new class of CDC-like toxins [114]. Furthermore, the positioning, orientation and molecular contacts of the F/Y-F/Y-Xn-YGR motif in the *Elizabethkingia anophelis* CDC-like toxin and PFO are conserved. This suggests a potentially key role for this motif in sensing and driving the transition from pre-pore to pore state [114]. Further structural studies of the fully formed CDCs pore will be expected to provide further detail on features that are required for the pre-pore to pore conversion.

CDCs play important roles in pathogenesis of the producing organism. During *S. pneumoniae* infection, PLY promotes apoptosis of macrophages and neural cells and stimulates production of cytokines and activation of the NLRP3 inflammasome, causing inflammation [115]. Furthermore, PLY can initiate the formation of neutrophil extracellular traps (NETs) and trigger the complement cascade [115, 116]. Both strategies represent examples of how *S. pneumoniae* can hijack the host defence to promote its infection. Recruitment of complement proteins by secreted PLY is thought to reduce the amount of available complement proteins that can bind and recognise *S. pneumoniae* cells [115]. Similarly, PFO mode of action also involves activation of the NLRP3 inflammasome and cytokine release. PFO-mediated NLRP3 inflammasome activation was demonstrated to be a key factor in causing necrosis of muscular tissue during *C. perfringens *-mediated gangrene [117]. SLO contributes to *S. pyogenes* evasion of the immune system by promoting apoptosis of macrophages and neutrophils [118]. A later study found that SLO promotes *S. pyogenes* virulence by inhibiting the protective activity of neutrophils. Similar to PLY, SLO inhibits formation of NETs and the oxidative burst, enabling its survival in the bloodstream [119]. Furthermore, an oxygen-stable version of SLO (SLS), also produced by *S. pyogenes *, promotes degradation of glycogen synthase kinase-3β in a ubiquitin/proteasome dependent manner, leading to macrophage cell death [120].

LLO, similarly to other CDCs, mediates apoptosis of immune cells and lymphocytes [121]. Additionally, *L. monocytogenes* can hijack host cell signalling pathways, cytokine production and activation of different inflammasomes through LLO-mediated pore formation [121]. Interestingly, LLO is fundamental for both formation of *Listeria *-containing phagosomes during chronic infection and their subsequent release through pore formation at the phagosome membrane. Furthermore, LLO can also promote organelle damage, reactive oxygen species (ROS) production and autophagy, highlighting the complex role that this single toxin can play in infection established by *L. monocytogenes* [121].

#### The RTX toxins

Repeat-in-toxin (RTX) toxins are produced by many different Gram-negative bacteria and include the adenylate cyclase (CyaA or ACT) of *Bordetella pertussis,* α-haemolysin HlyA secreted by uropathogenic *E. coli *, and leukotoxins LtxA and LktA secreted by *Aggregatibacter actinomycetemcomitans* and *Mannheimia haemolytica *, respectively (Table 1). The RTX family also includes the multifunctional autoprocessing repeats-in-toxin (MARTX) mainly produced by *Vibrio* and *Aeromonas* spp [19, 122] (Table 1). Among the RTX toxins, CyaA is the most well characterised, and is discussed in detail below.

**Fig. 4.**
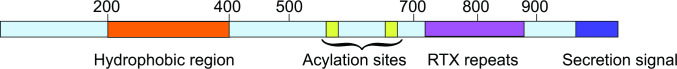
Schematic representation of the domain organisation of RTX toxin HlyA.

RTX toxins share a common motif that contains the nonapeptide repeat G–G–X–G–(N/D) –D–X–(L/I/V/W/Y/F)–X, which forms a β-roll and is involved in Ca^2+^ binding [19, 123]. Binding to Ca^2+^ was shown to be important for folding of some RTXs [124], although a role for the metal in the process of pore formation is not yet clear, as both HlyA and CyaA form pores in lipid bilayers in the absence of Ca^2+^ [125, 126]. RTX loci normally encode the RTX toxin (RtxA), an acyltransferase (RtxC), and three additional proteins, RtxB, RtxD, and RtxE, which assemble to form a T1SS and are responsible for toxin secretion [19]. RTX toxin monomers are normally large multidomain proteins. Typical RTX toxins contain a hydrophobic domain close to the N-terminus, which is responsible for pore formation, an activation domain that is the target of acylation, the Ca^2+^-binding repeat-containing motif and a secretion signal at the C-terminus (Fig. 4) [19, 127]. Fatty-acylation of HlyA has been shown to be central to promoting its oligomerisation, most likely by promoting structural rearrangements of the monomer [54, 128].

The CyaA domain organisation is unique, as its N-terminal domain carries its own enzymatic activity (adenylate cyclase) that, in addition to the pore-formation ability of the toxin results in the hijack of host cell functions through modulation of the second messenger cyclic AMP (cAMP) [19, 127].

Despite their early discovery, to date structural information of either RTX monomers or assembled pores is quite limited. An X-ray structure of the N-terminal domain of CyaA bound to host calmodulin has been solved [129], but structures of the full-length monomeric toxin or assembled pore are yet to be described. Structure determination will be critical to establish finally whether these toxins are α- or β-PFTs. To date, RTXs have been mainly studied using biophysical approaches. Experiments with lipid bilayers and patch clamping have shown that RTX pores have high selectivity for cations, and for HlyA and CyaA, an estimate of pore size has been determined [19, 54, 127]. In both cases, the pores are of small diameter and, consistently, examination of purified CyaA by native gel electrophoresis suggests formation of a dimer [130]. The biggest advance in understanding pore formation has been provided by AFM studies conducted on CyaA in proteoliposomes [131]. Membrane lesions could be generated in the presence of CyaA monomers (appearing as single dots on the surface of proteoliposomes), CyaA- arcs (incomplete pores) or CyaA fully formed large toroidal pores [131]. In the same study, blue native (BN)-PAGE analysis of CyaA in lipid vesicles confirmed the presence of several oligomeric forms [131].

Membrane lesions determined by CyaA were shown to increase in diameter over time, allowing passage of larger molecules, such as FITC-Dextran 20 kDa, suggesting that RTX pores have a large diameter, similar to CDCs [131]. CyaA monomers have only a small diameter [131]. This suggested that whilst the soluble monomers might fold into a compact structure, they will likely undergo structural changes to transition to an extended state, a finding consistent with observations from other PFTs. Using AFM it was also possible to observe that the height of RTX pores decreases from monomeric pores to the fully formed multimer. This allowed the authors to propose a model whereby monomers bind to the membrane, either by electrostatic interaction or specific interaction with a receptor, and subsequently undergo conformational changes to reach their extended state. Monomers insert into the membrane and are sequentially added to the forming pore, indicating that oligomerisation and membrane insertion occur in parallel, a feature that is typical of α-PFTs. This model is supported by the observation that increased toxin concentration and increased incubation times with lipid bilayers *in vitro* promoted the formation of pores with larger diameters [19, 131].

Many biophysical properties of RTX pores, including their diameter, are conserved across the toxin family [132]. Thus, it is likely that other RTX toxins will share many of the structural features observed for CyaA. Future studies of additional RTX toxin family members will reveal whether these possess a conserved fold and share a similar mechanism of assembly.

RTX toxins, similarly to other PFTs, cause the uncontrolled efflux of K^+^ and influx of Ca^2+^. Consequently, these changes interfere with the normal cellular inflammatory and immune responses. In the case of HlyA, this results in caspase-1 cleavage and IL-1β maturation. Furthermore, HlyA can cause de-ubiquitination of the NLRP3 inflammasome and mitochondrial dysfunction, ultimately leading to cell death [133]. HlyA additionally promotes proteolysis of several host proteins involved in adhesion, stimulating invasion [134]. In renal epithelial cells HlyA induces production of granulocyte-macrophage colony-stimulating factor (GM-CSF), thereby driving accumulation of M1 macrophages and leading to kidney injury [56].

CyaA represents a major virulence factor of *B. pertussis *, the bacterium that causes whooping cough. CyaA-specific targeting of myeloid cells expressing CD11/CD18 allows *B. pertussis* to efficiently hijack the immune response. CyaA-mediated pore formation causes influx of Ca^2+^ and efflux of K^+^ ions, potentially leading to several cellular responses, including initiation of inflammatory responses [135, 136]. However, many studies highlighted that the extent of CyaA role in subversion of immune response is mainly exerted by the adenylate cyclase domain, which is absent from other RTX toxins. This domain binds to calmodulin, initiating uncontrolled conversion of ATP to cAMP, subverting several signalling pathways. In phagocytes this results in loss of the oxidative burst, impairment of phagocytosis and increased secretion of immunomodulatory cytokines [24, 127]. Uncontrolled cAMP production additionally results in promotion of apoptosis in macrophages [137] and subversion of macrophage gene expression, disrupting the regulation between pro-inflammatory and immunoregulatory responses [127, 138–140]. Increased production of cAMP additionally leads to inhibition of the neutrophil’s ability to perform the oxidative burst and prevention of NET formation [141].

#### The VopQ toxin

VopQ is a pore-forming toxin found in *Vibrio* spp. that is secreted through the T3SS [26]. A combination of fluorescence microscopy and electrophysiology studies showed that VopQ can form pores of approximately 18 Å in lipid bilayers, which allows flux of small molecules up to around 400 Da [26]. Consistent with these findings, ectopic expression of VopQ was able to prevent lysosome acidification, and the protein was shown to interact with the vacuolar-type H^+^-ATPase (V-ATPase) V_0_ subunit at the vacuole, lysosome, endoplasmic reticulum or Golgi membranes [142]. These findings highlight that VopQ mode of action may be more complex, with potentially further roles in addition to pore formation.

More recently, the cryo-EM structure of VopQ bound to the V_o_ subcomplex of the V-ATPase has been solved [143]. Molecular contacts involve hydrophobic interactions between the membrane-embedded region of the V-ATPase with the VopQ transmembrane helices (TMHs) and electrostatic interactions between the V-ATPase cytoplasmic region and the VopQ C-terminal domain [143]. The residues involved in these interactions are highly conserved in V-ATPases across species, allowing VopQ to target both yeast and mammalian cells [143]. Furthermore, the structural study from Peng *et al.* highlighted that VopQ is mainly an α-helical protein, with three TMHs, potentially placing VopQ in the α-PFT class [143]. Whilst it was proven that VopQ undergoes conformational re-arrangements to transition from its soluble form [26, 143], to the membrane-inserted pore, the structure of its soluble form has not yet been solved. Thus, the extent of these structural re-arrangements remains to be elucidated.

Intriguingly, the three membrane-inserted TMHs are enriched in charged residues and are in contact with the membrane hydrophobic environment, thus destabilising the membrane and allowing leakage of ions [143]. This feature makes VopQ’s mode of action unique for bacterial toxins, as in other PFTs hydrophobic residues are in contact with lipids and surround the hydrophilic channel.

Additional modelling predictions performed using the VopQ structure and the structure of the other subcomplexes of the V-ATPase allowed Peng *et al.* to propose a model for VopQ’s multifaceted mode of action, whereby the protein forms a gated channel that prevents lysosome/vacuole acidification by forming outward gated pores, in agreement with previous findings [26, 143, 144]. Furthermore, binding of VopQ to the immature V-ATPase V_o_ subcomplex at the ER membrane sequesters V_o_ and prevents proper assembly of the full V-ATPase [143]. This, in turn, has a negative effect on membrane fusion between vacuoles and lysosomes [26, 144], leading to autophagy [144] and preventing *Vibrio parahaemolyticus* phagocytosis [145]. In response to VopQ-mediated intoxication, several cellular responses have been observed. Macrophages were shown to activate NOD-like receptor CARD domain-containing 3 (NLRC3) inflammasome and downregulate the activation of NOD-like receptor CARD domain-containing 4 (NLRC4) inflammasome complex [146, 147]. In Caco-2 cells VopQ invoked production of pro-inflammatory cytokine IL-8 by activating the mitogen-activated protein kinase/extracellular signal-regulated kinase (MAPK/ERK) pathway [148]. A recent metabolomic study in the Caco-2 cell line further showed that VopQ-mediated intoxication subverted glycolysis and energy metabolism, reducing amino-acid production and causing oxidative stress, collectively reducing survival of intoxicated cells [147].

#### T5SS-exported haemolysins

The class of haemolysins exported by the T5SS includes several distinct PFTs that show no similarity to other known classes (Table 1). The best characterised members of this group are ShlA, produced by *Serratia marcescens* and ExlA, secreted by *Pseudomonas aeruginosa* (Table 1). The structural features of these toxins as well as in-depth analysis of their mode of action and assembly remain largely elusive. The pore-forming domain of these toxins is localised at the C-terminal end [29, 149].

Although structural information on ShlA and ExlA pores are currently unavailable, biophysical characterisation of these PFTs has elucidated some aspects of the pore-formation mechanism. Investigation of ShlA pore formation in vesicles of different lipid composition showed that ShlA exhibits a preference for negatively charged lipids and that its pore-formation is dependent on phosphatidylserine [149]. More recent work on ExlA confirmed the importance of negatively charged lipids in the pore-formation mechanism and further demonstrated that ExlA binds to lipid rafts to increase its local concentration [150].

SAXS and NMR studies showed that in solution ExlA behaves as a molten globule, a characteristic that is typical of proteins that exist in multiple oligomeric and folding states [150]. Furthermore, AFM time-lapse analysis, conducted on ExlA-reconstituted liposomes, demonstrated that ExlA forms pores of various diameters, in a time-dependent manner [150]. A similar feature was also shown for ShlA [29]

Early studies conducted on a truncated form of ShlA lacking its C-terminal domain suggested that it exists exclusively in monomeric form in solution, and that single monomers insert into the membrane of red blood cells wherein they oligomerise [151]. However, subsequent structural analysis of the truncated form of HpmA, an ShlA homologue, suggests that this toxin can exist in solution in an oligomeric form [152]. The authors further identified several dimer forms with different interfaces and distinct putative stabilities [152]. It was further suggested that the most stable HpmA dimeric interface is involved in the cooperative activation of other, inactive HpmA toxins, facilitating template-assisted haemolysis [152, 153].

In addition to causing cellular lysis [154], ShlA can promote blebbing and autophagy of ocular epithelial cells [155, 156]. In non-phagocytic epithelial cells, ShlA mediates extracellular induction of autophagy, likely to promote bacterial intracellular survival [157]. Both ExlA and ShlA pores result in an increased Ca^2+^ influx in target cells, which in turn activates ADAM10. This metalloprotease cleaves E- or VE-cadherin, disrupting the cell-cell junctions of epithelial and endothelial cells [158]. In macrophages, the increased K^+^ efflux, mediated by ExlA, was shown to cause activation of the NLRP3 inflammasome, Caspase-1 activation and pyropoptosis [159].

## Anti-bacterial pore forming toxins

It is becoming increasingly evident that bacterial PFTs also play important roles in targeting other bacteria, allowing the producer to gain a fitness advantage in poly-microbial environments.

Bacteria have evolved competitive strategies to secrete harmful effector proteins (or toxins) and small metabolites that can intoxicate competitor microbes [1]. Effector proteins include large polymorphic toxins, which contain additional regions within the protein that aid secretion and delivery of the toxic, C-terminal domain or small toxins that are delivered directly into target cells (Table 2) [2–8]. In several cases, anti-bacterial effectors were found to harm competitor bacteria through formation of pores [160–163]. When secreted, anti-bacterial PFTs disrupt the integrity of the cytoplasmic membrane of target microbes. This causes leakage of ions, water and nutrients across the membrane barrier, a process that ultimately leads to protonmotive force (PMF) dissipation and ATP depletion.

Like the secretion of anti-eukaryotic PFTs, secretion of anti-bacterial PFTs is also mediated by specialised bacterial secretion systems. Some PFTs are delivered by the type V secretion system (T5SS), in particular by the two-partner contact-dependent inhibition (CDI) T5SS system [164]. The type VI (T6SS) and type VIIb secretion systems (T7SSb) appear to be somewhat specialised for anti-bacterial competition, and secrete numerous anti-bacterial toxins, including PFTs [165–167]. In the case of the T6SS, toxins are delivered directly into target cells [165], while for T7SS, the exact details of toxin delivery are still being investigated.

One peculiar method of toxin release is represented by some bacteriocins such as colicins, including those that exhibit pore-forming activity. Here, the colicin-encoding locus includes a lysis gene and, upon its induction, its product causes lysis of the producing cell and release of colicin toxin into the extracellular environment [163].

Anti-bacterial toxins, including PFTs, are usually are encoded alongside one or more genes coding for cognate immunity proteins [163, 165, 166]. Immunity proteins exhibit high specificity for their corresponding toxin and neutralise its activity, often (although not always) through direct binding [160, 162]. Immunity proteins are located at the cellular compartment where the cognate toxin exerts its function, thereby promptly preventing self-intoxication [160, 161, 163, 165].

### Receptors for anti-bacterial pore forming toxins

Due to the complexity of bacterial cell envelopes, many anti-bacterial PFTs depend on a receptor in the Gram-negative outer membrane to facilitate their import. This has been particularly well characterised for colicins (Table 2) [163]. For example, colicin N binds LPS with high affinity (Table 2), with efficient binding to both the oligosaccharide and lipid components [168]. The authors proposed that this interaction places colicin N in close proximity with outer membrane protein OmpF, which is the component that mediates its translocation [168, 169]. More recently it was demonstrated that OmpF is the primary receptor for colicin N as well as its translocator, but that LPS enhances the binding between the OmpF external surface and the colicin N globular domain [170]. Similarly, the colicin-like toxin pyocin S5 was recently demonstrated to interact with LPS-bound common polysaccharide antigen (CPA) as its primary receptor (Table 2) [162]. The full list of known outer membrane receptors for colicin PFTs are summarised in Table 2.

Anti-bacterial PFTs that are secreted by the T6SS do not require an outer membrane receptor because the secretion system directly delivers them into the periplasmic environment of target cells [165]. However, regardless of the routes by which PFTs reach the periplasm of target bacteria, they ultimately must insert into the cytoplasmic membrane to exert their toxic activities. To date mechanisms that initiate and drive pore formation are unknown, and it remains to be elucidated whether any host cell factors are required for this process.

### Prokaryotic-targeting toxin groups

With the exception of pore-forming colicins, which have long been studied [3], many anti-bacterial PFTs have only very recently been described. Consequently, mechanistic details of their assembly and membrane insertion processes are not yet known and it is, therefore, not possible to group them in families. For the purpose of this review, anti-bacterial toxins discussed in the following sections are grouped according to the specialised secretion system that mediates their delivery.

For each group of PFTs, where information is available, their structural features, assembly process and biophysical properties, including ion selectivity, are reviewed. Furthermore, their impact on target competitor bacteria and interaction with immunity proteins are also discussed.

#### Colicins

All characterised pore-forming colicins are α-PFTs, and are produced primarily by *E. coli* but also by *Citrobacter freundii* and *S. marcescens.* Colicins are released into the environment by producing bacteria upon their lysis and interact with a receptor, usually a protein, in the outer membrane of target cells. Following receptor binding, colicins subsequently interact with either TolA or TonB for import across the outer membrane. Colicins that use the Tol pathway for import are classified as group A, and those that use Ton are group B (Table 2) [163]. At present, any of the receptors utilised by pore-forming colicins to accumulate at the cytoplasmic membrane surface and initiate pore formation have yet to be reported.

Colicins are usually synthesised with a cognate immunity protein that protects sibling cells within the same population. For pore-forming colicins, immunity proteins are normally localised in the cytoplasmic membrane [163]. To date, several pore-forming colicins have been identified (Table 2), and there is structural information for some of these, but the exact architecture of assembled colicin pores remains unknown.

**Fig. 5.**
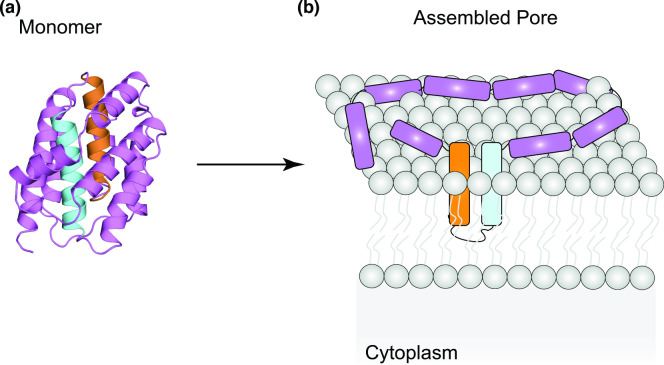
(**a**) Structure of the soluble monomer of colicin A (PDBID: 1COL). The pair of hydrophobic helices are depicted in cyan and orange. (**b**) Schematic depiction of the ‘umbrella model’ of colicin pore-formation, adapted from Cascales *et al.* (2007) [158]. Here, the pair of hydrophobic helices (in cyan and orange) are inserted in the membrane to constitute the pore, while the remaining helices lie on the membrane surface. Colicin oligomerisation and/or involvement of lipids as structural elements are then employed to form the final, active pore.

The first high-resolution structure of a colicin pore-forming domain was that of colicin A [171]. It comprises a bundle of eight amphipathic α-helices, surrounding a central hairpin formed by two hydrophobic α-helices (Fig. 5a) [171]. This deca-helical bundle arrangement is conserved across other pore-forming colicins and the colicin-like pyocin S5 [162, 172–176]. Pore-forming klebicins also show anti-bacterial activity and sequence similarity to characterised pore-forming colicins, implying that they also exhibit a similar fold and mode of action [177]. The packed arrangement of the hydrophobic α-helices within colicin monomers hints that, similar to other pore-forming toxins, substantial rearrangement takes place to promote pore insertion into the membrane.

Whilst a high-resolution structure of the formed pore is not yet available, two different models have been proposed for colicin pore assembly. The ‘umbrella model’ (Fig. 5b) [163, 178], supported by fluorescence resonance energy transfer and NMR studies [163, 178], posits that the two hydrophobic helices insert into the membrane, while the eight remaining helices rest on the membrane surface (Fig. 5b) [163, 178]. Subsequently, disulphide bond engineering experiments suggested an alternative ‘pen-knife model’ wherein the amphipathic helices rearrange, causing the two hydrophobic TMHs to ‘fall’ in the membrane [163]. A more recent study using site-directed spin labelling combined with EPR spectroscopy of colicin A inserted in proteoliposomes gave results that were more consistent with the umbrella model [179]. Colicin oligomerisation, following membrane insertion of monomers according to the umbrella model would be consistent with the assembly mechanism of other α-PFTs. Indeed, a subsequent study using similar biophysical approaches highlighted that colicin A pores exist as protein dimers [180]. Similarly, analysis of colicin Ia with negative-stain electron microscopy, following reconstitution into liposomes, suggested assembly of this toxin into a trimer of dimers [181]. While initial evidence indicated that colicin pores were monomeric [182], these recent findings, together with reports of colicin pores being permeable to large organic ions or exhibiting diameters up to ~10 Å, point instead to them being oligomeric assemblies [163, 183, 184]. In the case of colicin E1, formation of larger toroidal pores that involve lipids as structural components of the pore lumen has been proposed [185]. This model would provide an alternative explanation to the large lumens observed for certain colicin pores that are, however, thought to be monomeric. Obtaining higher resolution structural details for colicin pores would help to clarify the assembly model and oligomeric state.

All pore-forming colicins characterised to date form voltage-gated channels and their opening is promoted by positive voltages [163, 184, 186]. High voltage application could potentially trigger the conformational changes that promote colicin membrane insertion [187]. Interestingly, several reports have highlighted that the ion-selectivity of colicins is highly dependent on external conditions (i.e. pH, salt concentration, membrane lipid composition) [163, 185, 186, 188]. For instance, colicin E1 displays anion-selectivity, which is switched to cation selectivity when its conductance is assessed in anionic membranes [185, 188]. Furthermore, colicin B exhibits selectivity for cations, whereas colicin A shows a remarkably high preference for protons over any other cation [189, 190]. Conversely, colicin U produced by *Shigella boydii* does not appear to present such high selectivity [184]. It is possible that differences in amino acid composition in the pore-forming domain may contribute to the development of distinct selectivity filters.

Colicins and colicin-like toxins are part of a large grouping of bacteriocins, which include several additional classes of anti-microbial peptides, found both in Gram-positive and Gram-negative bacteria. While some bacteriocin peptides employ pore-formation as their anti-microbial strategy, these have recently been reviewed elsewhere [191] and will not be covered here.

#### Contact-dependent inhibition (CDI) proteins with pore forming activity

Proteins involved in contact-dependent inhibition (CDI) belong to the CdiA/CdiB family, wherein the specific toxic activity is found in the C-terminal domain of CdiA [192]. The enzymatic activity of CdiA-CT can vary across different toxins and species [164]. To date, only CdiA-CT^EC93^ secreted by *E. coli* EC93 was shown to dissipate the PMF of susceptible cells, additionally causing their metabolic shutdown [193]. Consistently, CdiA-CT^EC93^ reduced the ΔpH component of the PMF, possibly suggesting formation of a pore with selectivity for protons as its mode of action [193]. Since their discovery in *E. coli* EC93, CDI systems have been found in several other Proteobacteria and for many, the mode of action of their toxic C-terminal domain remains unknown [164, 192]. The structure and stoichiometry of CdiA-CT^EC93^ and mechanism of ion-selectivity are questions that remain to be addressed. As new CDI systems continue to be discovered it will be interesting to see whether additional CdiA toxins adopt a pore-forming mechanism of action.

#### T6SS-delivered pore forming toxins

To date, several unrelated pore-forming effectors have been characterised as secretion substrates of the T6SS [161, 194–196]. VasX, secreted by *V. cholerae *, exhibits structural homology with pore-forming colicins [197]. Consistent with this, VasX was shown to dissipate the membrane potential and disrupt membrane integrity of target *V. cholerae* strains during intraspecies competition [194, 197]. Recently, Tme1 and Tme2, secreted by *V. parahaemolyticus* were reported to exhibit a similar membrane-disrupting effect [196].

The anti-bacterial activity of VasX requires an accessory protein, VasW, likely involved in toxin secretion, and is relieved by its cognate immunity protein, TsiV2 (Table 2). Interestingly, VasX was also shown to play a role in virulence towards *Dictyostelium discoideum* [194]. Further characterisation of VasX, including determination of its pore-forming ability with vesicles constituted by different types of lipids could provide further information on the trans-kingdom activity of VasX, and whether it is determined by a promiscuous electrostatic binding to lipid-containing regions of target cells.

Unlike VasX, both Tse4 and Ssp6 show no sequence or structural homology to known colicins or to one another [161, 195], indicating that they form two novel families of T6SS-dependent PFTs. Structural information is not available for either toxin, but *in vivo* and electrophysiology studies have revealed some of the characteristics of these PFTs. Tse4 exhibits bacteriostatic activity against sensitive *P. aeruginosa* strains, which is relieved by the immunity protein Tsi4 [195]. Investigation of Tse4 mode of action highlighted that it dissipates the PMF of target cells by forming ion-selective pores, with high selectivity towards monovalent cations, but that the membrane integrity was not compromised [195]. Similarly, *S. marcescens* toxin Ssp6 also inhibits growth of target cells through PMF disruption, while leaving membrane permeability unaltered [161]. Additionally, Ssp6 was also reported to increase outer membrane permeability, although future work is required to determine whether this is directly caused by the toxin [161]. Consistently, the Ssp6 immunity protein, Sip6, localised to the outer membrane [161]. Cognate immunity proteins for anti-bacterial PFTs normally localise at the cytoplasmic membrane, likely to prevent assembly of the pore [163]. Sip6 localisation could represent a convenient means to interfere with PFT accumulation at the target membrane interface, whilst also preventing it from compromising outer membrane integrity [161].

*In vitro* reconstruction of Ssp6 activity demonstrated that the toxin formed pores that have a high selectivity towards monovalent cations, but do not allow flux of protons [161]. Interestingly, *in vivo* Tse4 characterisation revealed similar behaviour. Such selectivity towards specific monovalent cations would imply conserved amino-acid stretches or 3D fold to operate as selectivity filter [21]. Given the lack of sequence similarity reported between Tse4 and Ssp6 [161], resolution of the two PFTs structures could unveil whether they independently evolved distinct sequence features to operate as selectivity filters with similar ion-selectivity.

#### T7SS-delivered pore forming toxins

TspA is the first and only T7-dependent membrane depolarising toxin in *S. aureus* to be described to date [51]. TspA is a polymorphic toxin and is widespread in *Staphylococcus* spp., *Enterococcus* spp. and *Listeria* spp. TspA exhibits structural homology to colicin Ia and consistently, it showed the ability to depolarise but not permeabilise *E. coli* cells when heterologously produced, an effect that was relieved by its immunity protein TsaI (Table 2) [51]. These observations indicate that TspA might form ion-selective pores, similar to colicin Ia. Interestingly, TspA was demonstrated to exert anti-bacterial activity against *S. aureus* in a zebrafish model of bacterial competition [51]. Furthermore, TspA also contributed towards virulence in the same zebrafish model, and is therefore a potential a cross-kingdom effector [51]. Whether TspA activity represents a cooperative strategy to eliminate bacterial competitors and invade the host is still to be determined. Recent bioinformatic studies highlighted the presence of other T7SS-dependent effectors that display structural homology to colicin Ia [198, 199]. Structure determination of TspA and these predicted effectors could provide further insight on whether they adopt a similar 3D-structure or mechanism of pore-formation and, to define their selectivity.

### Concluding remarks

Pore-forming toxins are widespread in both Gram-positive and Gram-negative bacteria. They are additionally produced by higher organisms, such as Anemonae and Cnidaria [11, 200], indicating that they evolved quite early and represent an ancient attack form that, for bacteria, can be employed for both inter-bacterial competition and pathogenesis. While advances in the characterisation of PFTs are remarkable, many challenges and new questions remain open.

Purification of membrane proteins, including PFTs, is often challenging, with low yields and poor stability hindering structural studies [201]. The use of detergents and the introduction of styrene-maleic acid polymers (SMALPs), nanodiscs and amphipols, which mimic the membrane environment, have improved the overall stability of proteins and protein complexes. This has contributed to increased success of structural approaches, in particular cryo-EM analysis [202]. However, in the case of PFTs, additional challenges are posed by the highly dynamic nature of their membrane insertion process. Indeed, the complex structural rearrangements necessary for the transition from monomer to fully assembled pore has impeded full characterisation of the pore formation process [107, 202, 203]. Furthermore, members of several toxin families, such as CDC and RTX, often form arcs and incomplete pores, with the mixture of species further complicating structural resolution [107, 130]. AFM has been used successfully to investigate the dynamics of pore assembly for CDC and RTX PFTs [130, 204] and could be further employed in future studies with other families. More recently, the use of microfluidics devices for grid preparation in combination with time-resolved cryo-EM has shown incredible potential for studying dynamic conformational changes and heterogeneous structures [202, 205–208]. These approaches offer great promise for future study of the structural rearrangements necessary for PFT pore formation.

Structure-based drug design and antibody therapy have been successfully utilised for inhibition of several PFTs, through interfering with various stages of pore formation [209, 210]. Interestingly, several small molecules were found to inhibit Hla haemolytic activity by preventing its transition to the active pore [211, 212]. Similarly, flavonoids were demonstrated to bind PLY and LLO, preventing their oligomerisation and cytotoxicity [213, 214]. For Hla, several molecules were also found to target and irreversibly obstruct the assembled pore [215, 216]. In other studies, sequence- and structure-based design of small molecules has targeted PFTs through binding to their receptor-binding pocket [40, 217].

The clinical implications of understanding the structure and function of eukaryotic-targeting PFTs extends beyond combating microbial infections. Indeed, reports have shown that such PFTs can be engineered to target and kill cancerous cells [218, 219]. Furthermore, PFTs belonging to the CDC and haemolysin families have been employed to generate chimeric proteins with a controlled activation mechanism to efficiently deliver drugs to target cells [220–222].

The potential for anti-bacterial toxins to be engineered and utilised for therapeutical purposes has also been explored. A recent study showed that colicins can be used to disrupt biofilm integrity [223]. Moreover, when injected into the bloodstream of mice, pyocin S5 was observed to retain its functionality and to improve mouse survival to *Pseudomonas* infections [224]. Another study demonstrated that microcapsule delivery of the active form of colicin Ia reduced *E. coli* colonisation in murine models [225]. Engineering of chimeric proteins and use of cocktails of different toxins could provide a greater spectrum of activity and reduce the risk of resistance developing in target bacteria [226]. Similarly, in recent years peptidic bacteriocins have shown great promise for their efficacy as alternative anti-microbials when administered in model organisms [227].

Taken together, these reports highlight the great utility of engineering of colicins and, potentially, other PFTs for future therapeutic applications. As the molecular details of new PFTs emerge we anticipate this will provide scientists with further strategies to exploit them for clinical applications.
